# Narrative inquiry for CALL teacher preparation programs amidst the COVID-19 pandemic: language teachers’ technological needs and suggestions

**DOI:** 10.1007/s40692-022-00227-x

**Published:** 2022-03-09

**Authors:** Dara Tafazoli, Hussein Meihami

**Affiliations:** 1 School of Education, The University of Newcastle, Callaghan, Australia; 2 Department of English Language, Imam Khomeini International University, Qazvin, Iran

**Keywords:** Teachers’ technological needs, Technological pedagogical content knowledge (TPACK), CALL teacher preparation programs, CALL teacher professional development, Computer-assisted language learning (CALL)

## Abstract

Drawing on qualitative research, this study explores the Iranian EFL teachers’ technological needs and their suggestions for using computer-assisted language learning (CALL) during the COVID-19 pandemic. We analyzed the narratives produced by 66 English as a Foreign Language (EFL) teachers for their themes through deductive and inductive thematic analysis phases using MAXQDA. The findings indicate that CALL teacher preparation programs should prioritize technological pedagogical knowledge (TPK), then technological content knowledge (TCK), and finally technological knowledge (TK). Moreover, teachers stated that CALL teacher preparation programs should develop their knowledge concerning the intersections of TCK/TPK and TPK/TK. Furthermore, Iranian EFL teachers suggested that CALL can be used during the pandemic if cooperation among teachers, students, and parents will be made, technological-related infrastructures will be developed, and teacher preparation programs will develop EFL teachers’ collaboration, digital literacy, teacher autonomy, and TPK with special attention to the educational needs made by the pandemic situation. The findings have implications for teacher educators, professional development course designers and providers, and decision-makers by highlighting promising directions to devote their precious time and resources.

## Introduction and background

The COVID-19 outbreak has imposed a massive transformation on the education sector worldwide. The governments, including developed and developing countries, ordered the closure of all educational sectors to limit the spread of the virus. However, the circumstance is more confusing in developing countries (e.g., Iran), where the absence of resources makes fair and reasonable access arduous.

With an alarming rate of confirmed cases (6,019,947) and deaths (127,809) from January 3, 2020, to November 12, 2021 (WHO, 2021), Iran is an example of a developing country which is thoroughly impacted by this tragedy. In line with other countries and based on the WHO recommendations, all Iranian universities and schools have shifted to fully online education. Regardless of calling it an online, blended, or virtual education, Iranian educational sectors are experiencing ‘Emergency Remote Teaching’ (ERT) (Hodges et al., 2020), also called ‘emergency e-learning’ (Allen & Seaman, 2010) and/or ‘pandemic pedagogy’ (Milman, 2020). Tafazoli (2021a) believes that it would be wrong to call this provisional and conditional transition in the Iranian context ‘online education, while online education is based on prepared, well-chosen, and meticulous strategies in instructional design and curriculum. Considering the forward-looking effects of the pandemic, Iranian education should move away from a provisional ERT and conditional response to the forced situation and immigrate to a better quality of online education.

The global experiences showed that, apart from its opportunities, the transition from traditional and physical education to high-quality technology-based and online education is challenging. Therefore, the government, institutions, teachers, students, and parents, in close collaboration, should solve the challenges, including the acceptance and readiness of governments and institutions to move to online education (Bozkurt & Sharma, 2020), planning and modifying the policies regarding the accreditation of online education (Tafazoli & Atefi Boroujeni, 2021), technology-related psychological issues that affect teachers and students performance (Geng et al., 2019; Green et al., 2020), financial-related issues in equipping students and teachers with required resources (Aguilera-Hermida, 2020; Gillis & Krull, 2020), and teachers’ readiness and knowledge of effective technology-based teaching (Sepulveda-Escobar & Morrison, 2020; Tafazoli, 2021b).

The literature review showed that the same challenges are involved in the field of technology integration in language education, what is called Computer-Assisted Language Learning (CALL) (see Hong, 2010; Laabidi & Laabidi, 2016; Yeh & Swinehart, 2019), and the role of language teachers in dealing with these challenges is crucial. Several studies have acknowledged the positive effects of CALL on students’ achievements in speaking (Xie et al., 2021), writing (Abe, 2021; Reynolds & Kao, 2021), reading comprehension (Yang & Qian, 2020), vocabulary (Chen & Hsu, 2020; Li et al., 2021), willingness to communication (Lee & Drajati, 2020), interaction (Wrigglesworth, 2020), metacognition (Shih & Huang, 2020) attitudes (Webb & Doman, 2020), and motivation (Lamb & Arisandy, 2020). Despite its advantages in language learning, teachers are not interested in utilizing CALL in language teaching, which might be due to various reasons such as individual factors, contextual factors, and CALL teacher education and professional development (See Hong, 2010; Laabidi & Laabidi, 2016; Meihami, 2021; Tafazoli, 2021a; Yeh & Swinehart, 2019).

CALL teacher education and professional development is “a fundamental way of moving away from ERT and enabling university teachers to rethink their teaching practices and involve them in evaluating, planning, and applying educational technologies” (Tafazoli, 2021a, p. 5). CALL professional development (henceforth CALL PD) is recommended as a tool to modify the teachers’ beliefs, professional identity, and teaching philosophy (Dixon et al., 2014; Hur et al., 2016; Meihami & Esfandiari, 2021), motivate technology integration into teaching practices (Bataineh et al., 2020; Tafazoli et al., 2020), breakdown the teachers’ resistance and upskill them (Laabidi & Laabidi, 2016; Tayan, 2017; Van Gorp et al., 2019). However, some scholars (e.g., Anderson, 2012; Ball et al., 2008; Dashtestani, 2012, 2019) argue that lack of effective CALL PD has been among the most fundamental barriers to utilize technology in language classrooms. Besides the significance of teachers’ digital literacy, researchers highlighted the crucial role of pedagogy and content knowledge acclaimed by researchers as Technological Pedagogical Content Knowledge (TPACK) (Mishra & Koehler, 2006).

Although the eminence of TPACK is acknowledged and highlighted by many researchers (Bustamante, 2020; Harris et al., 2009; Janssen & Lazonder, 2016; Tafazoli, 2021a), studies in various countries have suggested that teachers do not have the capacity and cannot be expected to take full responsibility for integrating technology into schools (Gao et al., 2009; Hayes, 2007; Johnson et al., 2016; Player-Koro, 2013; Uluyol & Şahin, 2016; Ward & Parr, 2010). Also, available theoretical frameworks like TPACK are not discipline-based and do not provide teachers with sufficient detail on how to implement the required knowledge into a specific field (e.g., CALL) and might not be practically implementable in the teaching practices of a particular culture and context (Brantley-Dias & Ertmer, 2013; Koehler et al., 2015; Mouza, 2011).

Thus, the productive integration of technology in language education would not be practicable without proper CALL teacher preparation programs. However, CALL teacher preparation programs are partially unsuccessful due to the mismatch between the syllabus and teachers’ technological needs and priorities, notably in content and formats (Cosmah & Saine, 2013; Frontline Education, 2016), lack of detailed instruction (Lotherington et al., 2016), and not being available just in time (McCusker, 2017).

If teachers do not believe that a CALL teacher preparation program is not functional and helpful to address their professional needs, they are less interested in applying CALL tools and ideas in their teaching practices (Liao et al., 2017). However, due to the ever-changing nature of CALL, teachers’ technological needs, preferences, and technology-related syllabi and policies require varieties of preparation support (Martin et al., 2015). In other words, language teachers’ voice and authority in choosing the content and format will support the effective use of CALL in practice.

Therefore, we believe that teachers should take up CALL teacher preparation programs based on their needs in their immediate and educational settings. Thus, we should first assess and analyze the requirements and needs of CALL teacher preparation programs to be able to design, develop, and implement effective and successful CALL teacher preparation programs overall and for the pandemic situation. Hence, this study addressed the following research questions.

1. What are Iranian EFL teachers’ technological needs to be addressed in CALL teacher preparation programs?
2. What are Iranian EFL teachers’ suggestions for using CALL during the COVID-19 pandemics?

Before going through the study's theoretical framework (TPACK), we want to emphasize that, in this study, teachers’ technological needs refer to the general concept. In other words, we refer to what teachers need to be able to integrate technology in language teaching successfully.

## TPACK: reviewing the main concepts

**Fig. 1:**
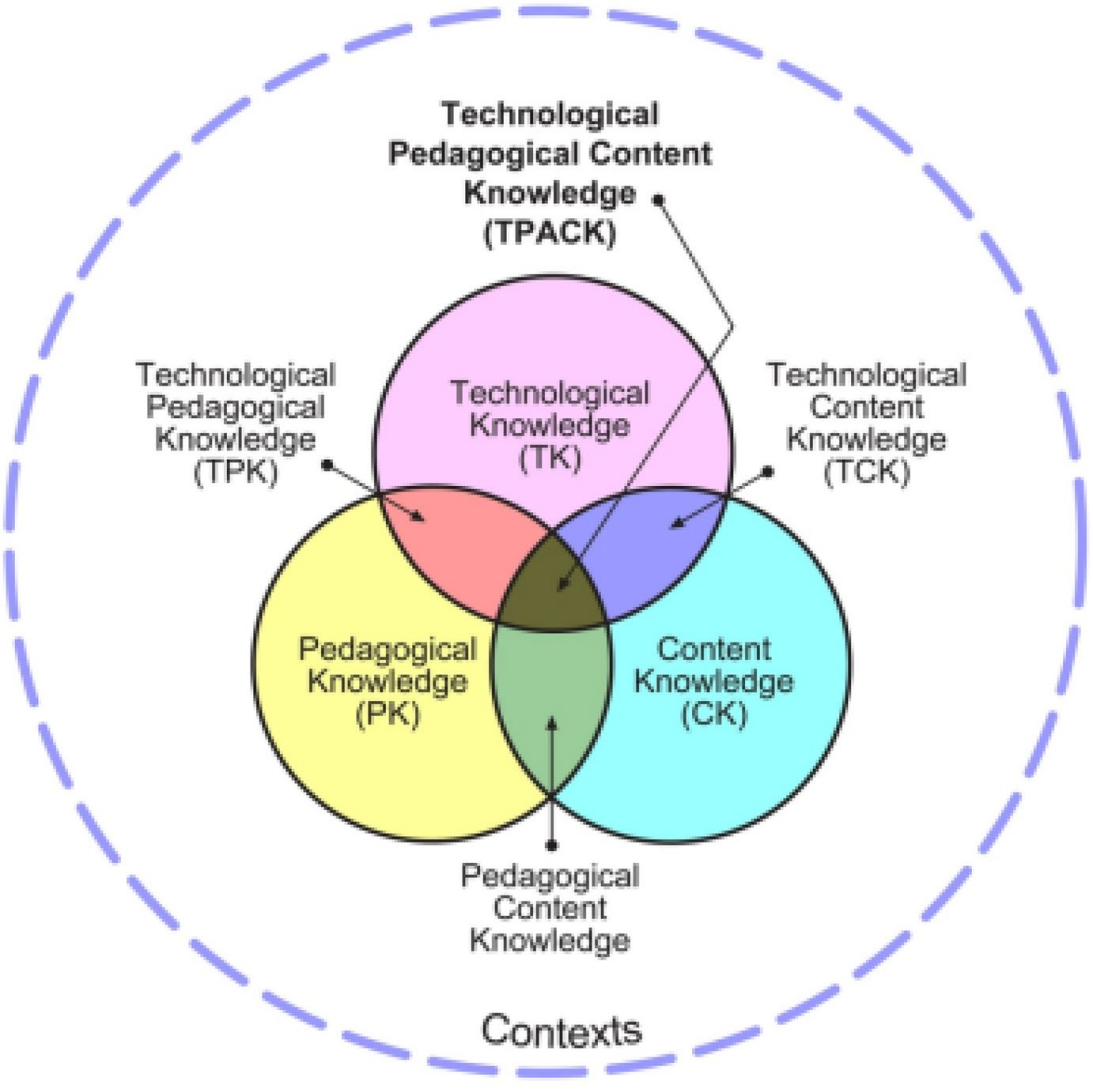
The TPACK Framework and its Knowledge Components (Extracted from Koehler & Mishra, 2009)

First introduced in 2006 by Mishra and Koehler, TPACK is a framework whose main purpose is to specify the nature of knowledge teachers need to integrate technology into their classes and, simultaneously, it attempts to address the complex multidimensional aspects of teacher knowledge. Although TPACK framework tends to emphasize the interplays among the three main forms of knowledge, including content, pedagogical, and technological knowledge, Koehler and Mishra (2009) stated that TPACK also emphasizes intersectional knowledge, including pedagogical content knowledge (PCK), technological content knowledge (TCK, technological pedagogical knowledge (TPK), and technological pedagogical content knowledge (See Fig. 1).

The theoretical descriptions of different types of knowledge emphasized in TPACK are given below:

- Content knowledge (CK): This type of knowledge refers to the subject matter knowledge which should be learned or instructed. The CK is pivotal for the teachers since it includes the knowledge of concepts, theories, established practices, and approaches (Shulman, 1986).
- Pedagogical knowledge (PK): This type of knowledge refers to approaches, methods, and teaching and learning techniques. Pedagogical knowledge focuses on how to teach, manage classrooms, plan lessons, and assess learners. According to Koehler and Mishra (2009), PK demands cognitive, social, and developmental theories on the part of teachers.
- Technological knowledge (TK): This is not easy to define and describe TK since each definition or description may be outdated over time. However, as Koehler and Mishra (2009) stated, TK can be “certain ways of thinking about and working with technology can apply to all technology tools and resources” (p. 64). Therefore, the important point concerning TK is that there is no “end state” for it since it is an evolving notion.
- Pedagogical content knowledge (PCK): Coined by Shulman (1986), PCK refers to the knowledge of pedagogy that can help teachers teach specific content. As described by Koehler and Mishra (2009, p. 64), “PCK covers the core business of teaching, learning, curriculum, assessment and reporting, such as the conditions that promote learning and the links among curriculum, assessment, and pedagogy.”
- Technological content knowledge (TCK): It is believed that technology and content of different subject matters have different facilitative and detrimental effects on one another. Therefore, TCK is to understand how technology and content can have effects on one another. Teachers need to know how content can be changed by using technology. Moreover, TCK is the knowledge through which teachers can understand which technologies can be appropriate to address the specific content of a subject matter.
- Technological pedagogical knowledge (TPK): This type of knowledge refers to the understanding of how using technology can change learning and teaching. According to Koehler and Mishra (2009), TPK includes the knowledge of pedagogical affordances and constraints of technologies in different pedagogical designs. When addressing TPK, it is rather critical to develop teachers’ awareness about the affordances and constraints of technological tools, so they can better decide which technologies to use with what pedagogical designs. This is critical for teachers since not all of the popular software and applications (such as Office Package) are designed for educational purposes, and teachers should be able to use them by developing their TPK.
- Technological pedagogical and content knowledge (TPACK): This type of knowledge emerges from the interaction among the three types of knowledge: content, pedagogy, and technology. Therefore, TPACK is different from the knowledge of all three concepts individually. Koehler and Mishra (2009) stated that:

TPACK is the basis of effective teaching with technology, requiring an understanding of the representation of concepts using technologies; pedagogical techniques that use technologies in constructive ways to teach content; knowledge of what makes concepts difficult or easy to learn and how technology can help redress some of the problems that students face; knowledge of students’ prior knowledge and theories of epistemology; and knowledge of how technologies can be used to build on existing knowledge to develop new epistemologies or strengthen old ones (p. 66).

Consequently, TPACK can be called upon in various situations to help teachers apply different technological solutions for different courses, teaching and learning methods, to mention a few. Thus, TPACK provides a flexible and pragmatic understanding for the teachers through which they can address technologies in unique situations.

## Methodology

### A narrative inquiry: a descriptive design

**Fig. 2:**
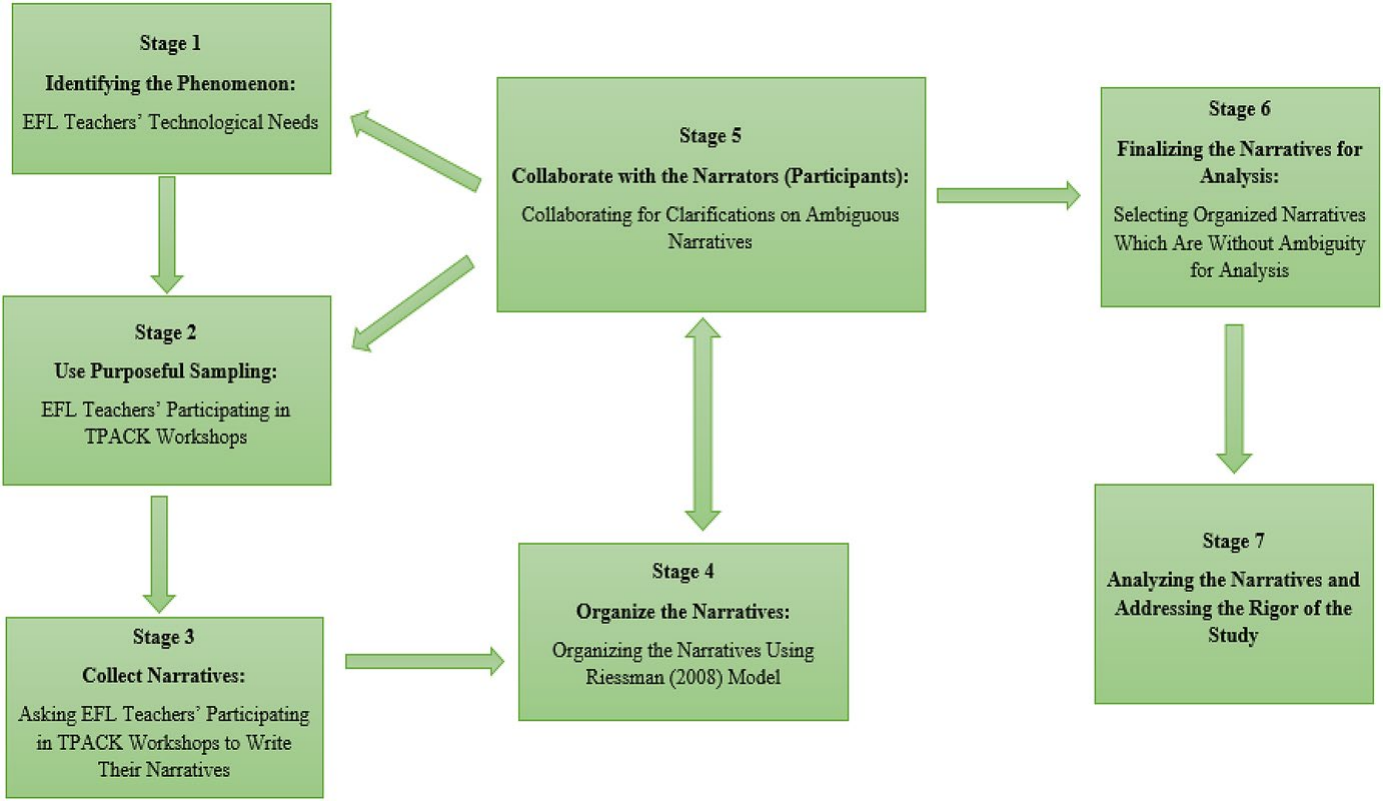
The descriptive narrative design of the study

This study addressed EFL teachers’ technological needs and their suggestions for using CALL during the COVID-19 pandemic through a narrative inquiry. Narratives can help researchers obtain information about a *phenomenon* (Creswell & Poth, 2018). Furthermore, through narratives produced by individuals, researchers can address different contextual issues to identify individuals’ perceptions (Riessman, 2008). Thus, in the current study, we adapted a descriptive narrative design proposed by Edmonds and Kennedy (2017) to reach the needs of Iranian EFL teachers and their suggestions for using CALL during the COVID-19 pandemic through their narratives. Figure 2 shows the descriptive narrative design of the study.

The descriptive narrative design is an appropriate design when the researchers describe certain conditions or phenomena concerning individuals. We went through seven stages to address the research design. First, we identified the phenomena to be explored using the current research design: The Iranian EFL teachers’ technological needs and their suggestions for using CALL in the COVID-19 pandemic. In the second stage, we purposefully selected Iranian EFL teachers who participated in the webinars. The reason behind this purposeful selection was that these teachers were aware of the overall concepts of technology in L2 pedagogy. Narrators’ awareness of the phenomenon is critical in narrative inquiry to obtain accurate and valid narratives (Czarniawska, 2004). In the third stage, then, we asked the participants to write their autobiographical narratives. In this stage, we posed two questions to help the EFL teachers direct their autobiographical memory (Smorti, 2011) toward their needs. The two questions related to the study’s research questions were “what are your most critical needs to be addressed in CALL professional development programs?” and “what are your suggestions for using CALL during the COVID-19 pandemic?” The first question directed their memories to produce their narratives concerning their needs, and the second question directed their narratives toward the needs they felt during the pandemic. In the fourth stage, we organized the narratives. In this stage, we used Riessman’s (2008) narrative model to select those narratives that could be analyzed. To do so, we selected the narratives which had an abstract, orientation (introduction), complicating action, evaluation, resolution, and coda. Riessman (2008) believed that a ‘fully-formed’ narrative that can provide the necessary information to investigate a topic should have six elements. Based on the model, the narrators need to summarize the main points of their stories in the abstract. They have to consider time, place, situations, and characters in their narratives to show the orientation of their stories. The narrators highlight the turning points and crises in theirs stories by addressing the events to address the complicating actions. Then, they need to evaluate their stories critically. This is the soul of the narratives, and it is very conducive to figure out the main points from the perspectives of the narrators. After addressing the resolution which is about the results of the events, they address the coda of their narratives by linking the past to the current time.

From 111 EFL teachers who wrote their narratives for us in this stage, we selected 66 narratives for further analysis. In the fifth stage, collaboration was done with the narrators of ambiguous narratives to clarify the issues. It is important to note that stage five is similar to a juncture in descriptive narrative design in which the researchers can revert to any of the previous stages indicated by arrows to rectify the probable issues. This is related to the emergent nature of this design, contributing to comprehensive data collection and analysis. Then, in the sixth stage, we finalized narrative selection: 66 narratives by Iranian EFL teachers. Finally, in the seventh stage, we went through a deductive-inductive thematic analysis to analyze the narratives. Moreover, we addressed the study’s rigor, namely credibility, transferability, and dependability (Ary et al., 2014).

### The webinars and participants

Due to the COVID-19 situation and the necessity of developing language teachers’ skills, the first author commenced a series of webinars titled ‘*Language teachers’ professional development amid the COVID-19*’. The webinars were conducted through Zoom as the primary medium, and the instructor used various tools such as Prezi, Focusky, Kahoot, wordle, multimedia tools, to mention a few, for better interaction and instruction. The webinars consist of various sections, including (a) conceptualizing online education, (b) the necessity of CALL, (c) challenges of online education, (d) merits and barriers of CALL, (e) CALL teacher education, (f) conceptualizing TPACK, (g) TPACK in practice, and h) Q&A sessions. The participants of this study were 66 Iranian EFL teachers who took part in the webinars. First, we asked 111 EFL teachers to write their autobiographical narratives in response to two questions concerning their needs, which could be addressed in CALL teacher development programs, and their suggestions for using CALL during COVID-19. However, just 66 narratives followed Riessman’s (2008) narrative model. As stated earlier, we filtered the narratives based on Riessman’s (2008) model because we wanted to reach “fully formed” narratives that were conducive to picture out the topic under study. However, this is critical to mention that we reviewed all of the narratives, both those that followed Riessman’s model and those which not, in case they might include valuable information. However, there were no important data in the narratives which did not follow Riessman’s model. This is logical since a narrative can provide relevant and valuable information when it follows a specified structure (Riessman, 2008). Otherwise, the amalgamation of different and irrelevant ideas makes it a tough task for the researchers to extract the main points. Table 1 shows the background characteristics of the participants.

**Table 1:** Background characteristics of the participants

Educational level	Gender	No	Age Mean	Years of overall teaching experience
BA in TEFL	Male	12	25	3
	Female	18	26	4
MA in TEFL	Male	10	31	6
	Female	20	33	8
PhD in TEFL	Male	1	42	9
	Female	5	40	11
Total	Male	23	32	6
	Female	43	33	8

As seen in Table 1, 30 participants (12 males and 18 females) had a BA degree in TEFL (Teaching English as a Foreign Language); 30 participants (10 males and 20 females) hold an MA in TEFL, and 6 participants (1 male and five females) hold a PhD in TEFL. Overall, 66 participants (23 males and 43 females) wrote their narratives and delivered them.

### Source of data: autobiographical narratives

The EFL teachers were asked to write their autobiographical narratives concerning their needs and their suggestions for using CALL during the COVID-19 pandemic. The prerequisite of producing autobiographical narratives is to activate autobiographical memories concerning an issue (Smorti, 2011). Thus, we posed two questions to direct EFL teachers’ memory concerning needs and their suggestions for using CALL to address L2 pedagogy during the COVID-19 pandemic. The narratives delivered by the EFL teachers had varying lengths, the smallest 150 words to the largest 650 words. We attempted to organize the narratives based on Riessman’s (2008) narrative model. Thus, we divided the narratives into abstract, introduction, evaluation, resolution, and coda to obtain as much information from them.

### Data analysis procedure: a deductive-inductive thematic analysis

**Fig. 3:**
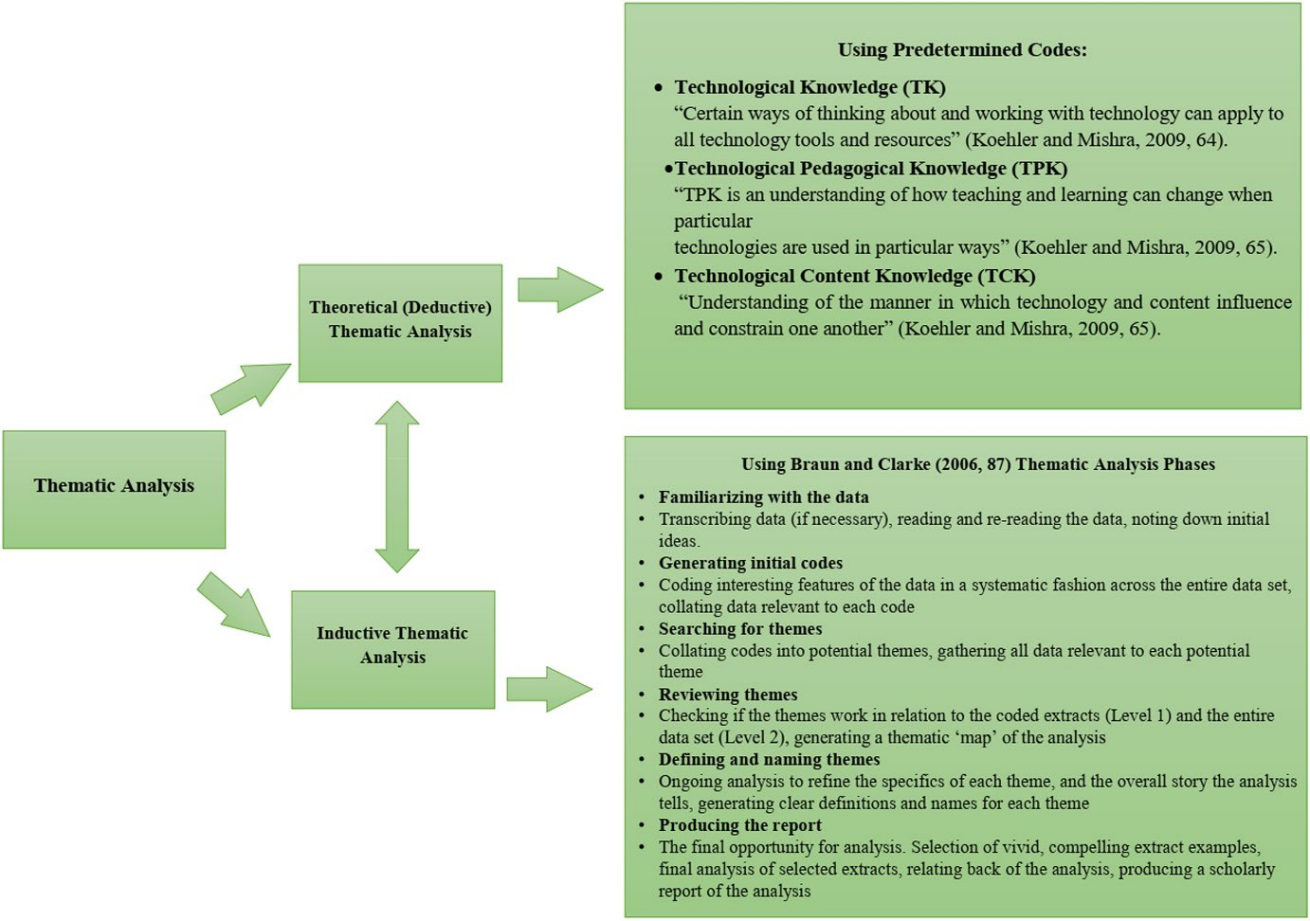
Thematic analysis procedure

Through thematic analysis, the researchers could reflect the reality (Braun & Clarke, 2006) of EFL teachers’ technological needs and their suggestions for using CALL during the COVID-19 pandemic. Therefore, we used a deductive-inductive thematic analysis. The reason behind selecting this type of thematic analysis was that the research questions in the current study were both explanatory and exploratory. While the first research question posed to obtain EFL teachers’ technological needs based on technological aspects of TPACK, including technological knowledge (TK), technological pedagogical knowledge (TPK), and technological content knowledge (TCK), used as predetermined codes (deductive thematic analysis), the second question delved into EFL teachers’ suggestions for using CALL during COVID-19 which required delving into the data inductively to find the main themes. The procedure through which we used the thematic analysis is shown in Fig. 3.

As shown in Fig. 3, we addressed the first research question concerning EFL teachers’ technological needs through deductive thematic analysis. To do so, we analyzed the narratives produced by the EFL teachers for their themes related to TK, TPK, and TCK. We put the narratives into MAXQDA and codified the content. We used the definitions of the three codes (TK, TPK, and TCK) as the criteria to codify the content. For instance, one of the teachers stated, “*teaching us how to make interesting technological materials for our learners*” which is related to TCK, the relationship between technology and creating L2 content.

We used inductive thematic analysis to address the second research question posed to understand EFL teachers’ suggestions to use CALL during COVID-19. To do so, we went through thematic analysis phases proposed by Braun and Clarke (2006), using MAXQDA. We went through different phases, recursively, to thematize the data. In the first phase, we put the narratives into MAXQDA and read and reread the initial ideas. In the second phase, we codified the data based on the initial ideas and semantically and latently revealing extracts (Braun & Clarke, 2006). While the semantically revealing extracts showed a code explicitly, the latently revealing extracts were obtained implicitly through examining the related literature. For example, when we reached utterances such as “[time is one of] *the barriers that are extrinsic to teachers*” or “enhance the required network infrastructures”, we formulated the first relevant ideas: the importance of infrastructures, internal–external supports, to mention a few. In the third phase, then, we collated the initial codes into potential themes. We did so with the help of Creative Coding of MAXQDA, enabling us to create potential parent codes and potential themes. In the third phase of data analysis, by using Braun and Clarke’s (2006) thematic analysis framework, we communicated the parent codes and potential themes with some narrators to see whether the themes and codes addressed their intentions. Practically, we merged the initial ideas by reviewing the literature, finding the associations among the initial ideas, and seeking the narrators’ ideas. For instance, the importance of time, the good internet connection, and user-friendly software are the ideas related to the development of infrastructures in CALL (Tafazoli, 2021a); therefore, we selected infrastructure development as a potential theme. In the fourth phase, we reviewed the themes concerning the entire data using Smart Coding Tools of MAXQDA, which helped us create a comprehensive thematic map after some modifications, deletions, and combinations were applied. In the fifth phase, we rechecked the names of the obtained themes to see whether they were in accordance with the entire data. Here, we used interesting and revealing words or phrases used in the EFL teachers’ narratives, if any, to rename the themes. Finally, in the sixth phase, we provided the analysis report by selecting some vivid and compelling extract examples shown in the Finding section.

### The rigor of the study

Addressing the rigor of the study is rather critical in qualitative studies to obtain robust findings. Thus, we addressed the credibility, transferability, and dependability (Ary et al., 2014) of the findings in the current study. To enhance the credibility of the findings (equal to internal validity in quantitative studies), we used the peer debriefing strategy in which a colleague of us, holding an MA in applied linguistics, went through the thematic analysis procedure for some parts of the data to see whether she could reach the same themes. Moreover, we enhanced the process of thematization through member checking in which we checked the themes that we obtained with some of the narrators. Furthermore, we addressed the transferability of the findings (equal to the external validity of quantitative studies) by using cross-case comparison. To do so, our participants were at different educational levels and teaching experiences. These participants were EFL teachers across different parts of Iran whose narratives could enhance the transferability of the findings. Finally, we addressed the dependability of the findings (equal to reliability in quantitative studies) through inter-coder agreement. To do so, another coder, who helped us deal with the credibility of the findings, codified 20% of the data. The inter-coder agreement was 90%. The inter-coder agreement was obtained through the Teamwork tools of MAXQDA.

## Findings

The findings of the study will be presented in the following paragraphs. First, we present the findings concerning the first research question about EFL teachers’ technological needs. Second, we present the findings concerning the second research question in which we saught EFL teachers’ suggestions for using CALL during the COVID-19 pandemic.

### EFL teachers needs: TK, TPK, and TCK

**Fig. 4:**
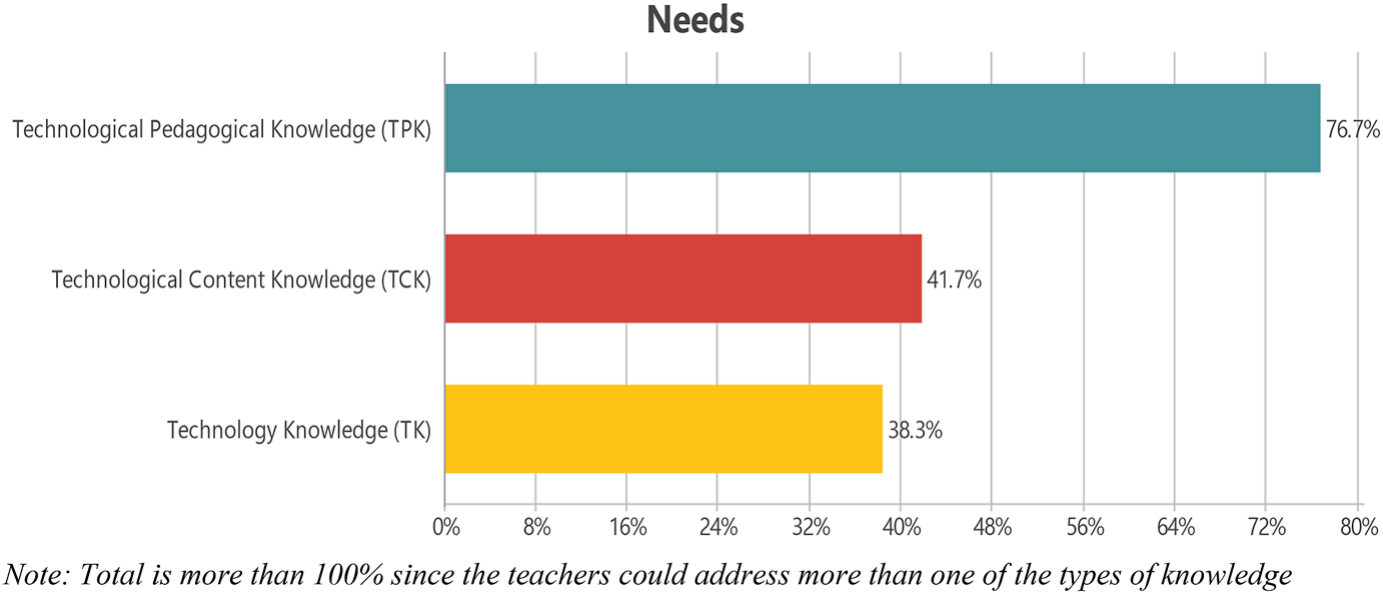
EFL teachers’ technological needs. *Note* Total is more than 100% since the teachers could address more than one of the types of knowledge

The analyses of narratives produced by Iranian EFL teachers indicated their needs concerning TK, TPK, and TCK. Figure 4 shows the percentage of TK, TPK, and TCK themes within the EFL teachers’ narratives.

As seen in Fig. 4, 76.7% of the EFL teachers asserted that CALL teacher preparation programs should develop their TPK. They believed that different aspects of TPK, such as classroom management, should be addressed in CALL teacher preparation programs. For example, a teacher asserted in his narrative that “*the classroom management is the issue and how it is different from face-to-face classes*” should be addressed in CALL teacher preparation programs to help them “*utilize new and creative methods of teaching English online*.” Developing pedagogical knowledge concerning technology “*is on top of the list*” for the EFL teachers, mostly when “[they] *are not very experienced, so* [their] *need is more practical information rather than just theories.*” EFL teachers also believed that CALL teacher preparation programs need to help them with pedagogical techniques concerning technology such as “*methods of testing online*.”

Figure 4 also shows that EFL teachers’ second critical need obtained from their narratives was TCK (41.7%). EFL teachers believed that CALL teacher preparation programs need to help them “*provide authentic materials for students … the materials must be interesting to students*”. Furthermore, they asserted that CALL teacher preparation programs should help them become autonomous teachers to work “*on how to make their lesson plans, adding more color to them by different apps/websites*.” Moreover, related to EFL teachers’ autonomy, they pointed out that CALL teacher preparation programs need to help them with the “*haphazard transition from traditional classrooms to virtual ones*” to produce “*creative content*.” EFL teachers believed that if creativity would be addressed in CALL teacher preparation programs, it could lead to “*innovative pedagogy*.”

The third need that EFL teachers asserted was TK (38.3%). They thought of CALL teacher preparation programs as a “*guide*” for the teachers to help them develop their knowledge about “*how to use the applications*.” Such a “*guide*” program needs to provide them with knowledge so that they can deal with “*the serious challenges of new technology types*” to “*improve* [their] *knowledge in the field of using technology and become familiar with modern technologies*.” Overall, the EFL teachers stated that one of the critical issues that should be addressed in the CALL teacher preparation programs is developing their TK to “*develop their TPK and TCK*.”

**Fig. 5:**
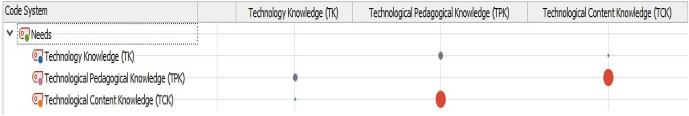
The relations among TK, TPK, and TCK

We found some relationships among the three needs through further analyses of the EFL teachers’ narratives with the Code Relations Browser: TK, TPK, and TCK. Figure 5 indicates the relationships.

As seen in Fig. 5, there is a strong relationship between TPK and TCK. The mechanism through which Code Relations Browser obtains such a relationship is the co-occurrence of the codes in a narrative. It shows that EFL teachers supposed that CALL teacher preparation programs need to address some issues which are simultaneously related to TPK and TCK. For instance, they believed that CALL teacher preparation programs should help them “*develop proper online material to satisfy the institute that* [they] *have to change* [their] *method*.” This is the intersection between TCK and TPK since the teachers should obtain knowledge to create technological knowledge for a specific pedagogical context. Furthermore, one of the teachers asserted that CALL teacher preparation programs should help us “[develop] *useful activities …in relation to classroom management*”, this is where TCK and TPK are merged.

Figure 5 shows a less strong relationship between TPK and TK than the relationship between TPK and TCK. This indicates that teacher preparation programs should develop their knowledge concerning TPK and TK so that they can use technology types with specific pedagogical needs. For example, they believed that CALL teacher preparations “*should provide us with new software or applications to use them in teaching*” so that they “*can come up with some specific teaching contexts and needs*.” They also asserted that “[we] *reckon we desperately need CALL Teacher Education to become familiar with different platforms and the different techniques to have a faultless class*.” Thus, this is rather important to provide a venue in CALL teacher preparation programs in which knowledge concerning the intersection of TPK/TK and TPK/TCK will be introduced to EFL teachers.

### EFL teachers suggestions for using CALL during COVID-19 pandemic

**Fig. 6:**
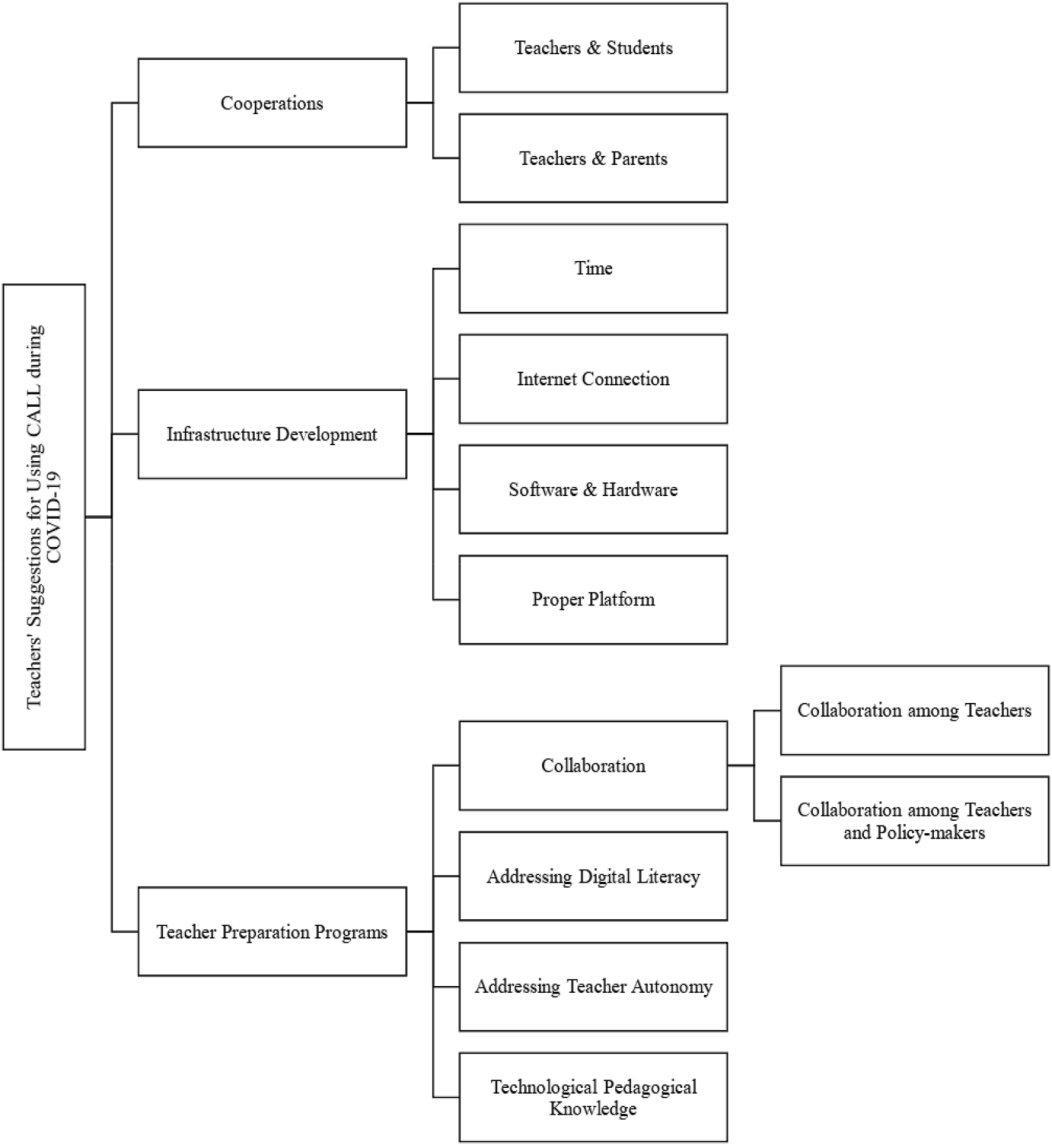
EFL teachers suggestions for using CALL during COVID-19 pandemic

Through conducting inductive thematic analysis on the narratives authored by EFL teachers, we obtained several main themes and sub-themes concerning their suggestions for using CALL during the COVID-19 pandemic. Figure 6 shows the themes and sub-themes in this regard.

The findings indicated three main themes related to EFL teachers’ suggestions for using CALL during the COVID-19 pandemic. The first theme obtained through the narrative analyses was cooperation among teachers, students, and parents. They asserted that using CALL during the pandemic needs to “*encourage students, their families, and all teachers to cooperate*” through “*having more webinars for students and teachers* [and families].” Moreover, they alleged that to use CALL during the pandemic needs to “f*ind out how parents can be in touch with the teachers of their children or school to stay informed, ask questions and get more guidance.”* Hence, *“parent groups or community groups can be a good way to support each other* [families] *with their children homeschooling*.” This shows that the cooperation among teachers, students, and parents can be conducive to deal with problems made by the pandemic for using CALL, such as “ *the lack of trust which is clearly a problem with students and/or their families*.”

The second theme addressed by the EFL teachers to use CALL during the pandemic was infrastructure development. The theme has four main sub-themes: time, internet connection, hardware and software, and proper platform. They assumed that insufficient time is “[one] *the barriers that are extrinsic to teachers*” and should be revisited through creating a “*new curriculum based on ICT use*.” Another infrastructure-related theme was the internet connection. EFL teachers stated that the government should “*enhance the required network infrastructures*” and “*support the teachers with the cost of the internet*.” More often than not, most EFL teachers claimed that “*the basic and the most important problem* [of using CALL] *is the internet connections!*” Furthermore, the EFL teachers believed that hardware and software development should be considered if the educational system wants to use CALL. They asserted that “*introducing effective and handy apps*” and “*available hardware*” can be conducive to using CALL during the pandemic.

They believed that teachers should have a “*sympathy*” feeling with the students who do not have access to the technological requirements. They also held that choosing appropriate software can motivate the learners. EFL teachers pointed out that “*the teachers should first know about the devices* [hardware] *and applications* [software] *which students use*, *to decide which platform they should use*.” Finally, selecting and using a proper platform can help magnify CALL’s applicability during the pandemic. As stated earlier, EFL teachers believed that appropriate platforms should be chosen after knowing which devices and applications students can use. They mentioned that the government should develop localized platforms since they had problems with the current global platforms; as they stated, “*Big Blue Button is not free, and LMS company must support it and using zoom needs VPN and use a lot of internet traffic*.” They stated that if a proper platform would be used, then the process of teaching and learning can be enhanced since “*online discussion panels can help* [all learners such as] *a shy person to start talking in a foreign language*.”

One of the themes obtained from the EFL teachers’ narratives was the role of CALL teacher preparation programs focusing on the EFL teachers’ technological needs during the pandemic. EFL teachers believed that they “*need CALL teacher educations and some technological-based webinars related to teaching*” on different issues of using CALL in L2 pedagogy such as “*how to work with e-learning platforms*,” “[how] *to move with global e-learning and make teaching efficient*,” and “[to] *accept that their approaches to learning in traditional classes are not working anymore.*” In addition, some of them considered CALL teacher preparation programs a venue for building collaboration among teachers and policymakers. They asserted that CALL teacher preparation programs could “*create groups in which teachers will be able to share their experiences*”; thus, by so doing, they can “*make a strong support team to solve technological glitches faced by the instructors and learners*.” Moreover, EFL teachers highlighted the role of CALL teacher education programs to develop digital literacy among EFL teachers to conquer the educational issues related to technology during the pandemic. They mentioned that CALL teacher education programs could develop digital literacy among EFL teachers to “*identify appropriate online tools for children and offer advice for age-appropriate apps, games and other online entertainment*” so that they can “*deal with upcoming difficulties in classes*.” Furthermore, developing their digital literacy may help them with materials selection, evaluations, and implementation.

The EFL teachers also believed that CALL teacher education programs could develop teachers’ autonomy to deal with the problems raised by the pandemic through “*activating teachers’ creativity and critical thinking*,” “*giving them the right to choose according to their own and their students’ needs and particular context requirements*,” and “*using creative teaching methods, …* [and] *online education software*”. Developing their autonomy in this way can help them identify the “*real needs of their learners in this hard time and try to be flexible and positive*.” Finally, they asserted that CALL teacher preparation programs should address specific TPK for the pandemic time through introducing “*teaching methods utilizing CALL principles*,” “*how to assess online classes*”, and “*how to implement different teaching methods and techniques in online classes*.” They also stated that CALL teacher preparation programs need to address the pedagogical knowledge to help teachers “*promote reflection and interaction, encourage each student to interact in the online class, create a supportive learning environment, and use learning tools for better engagement*.”

**Fig. 7:**
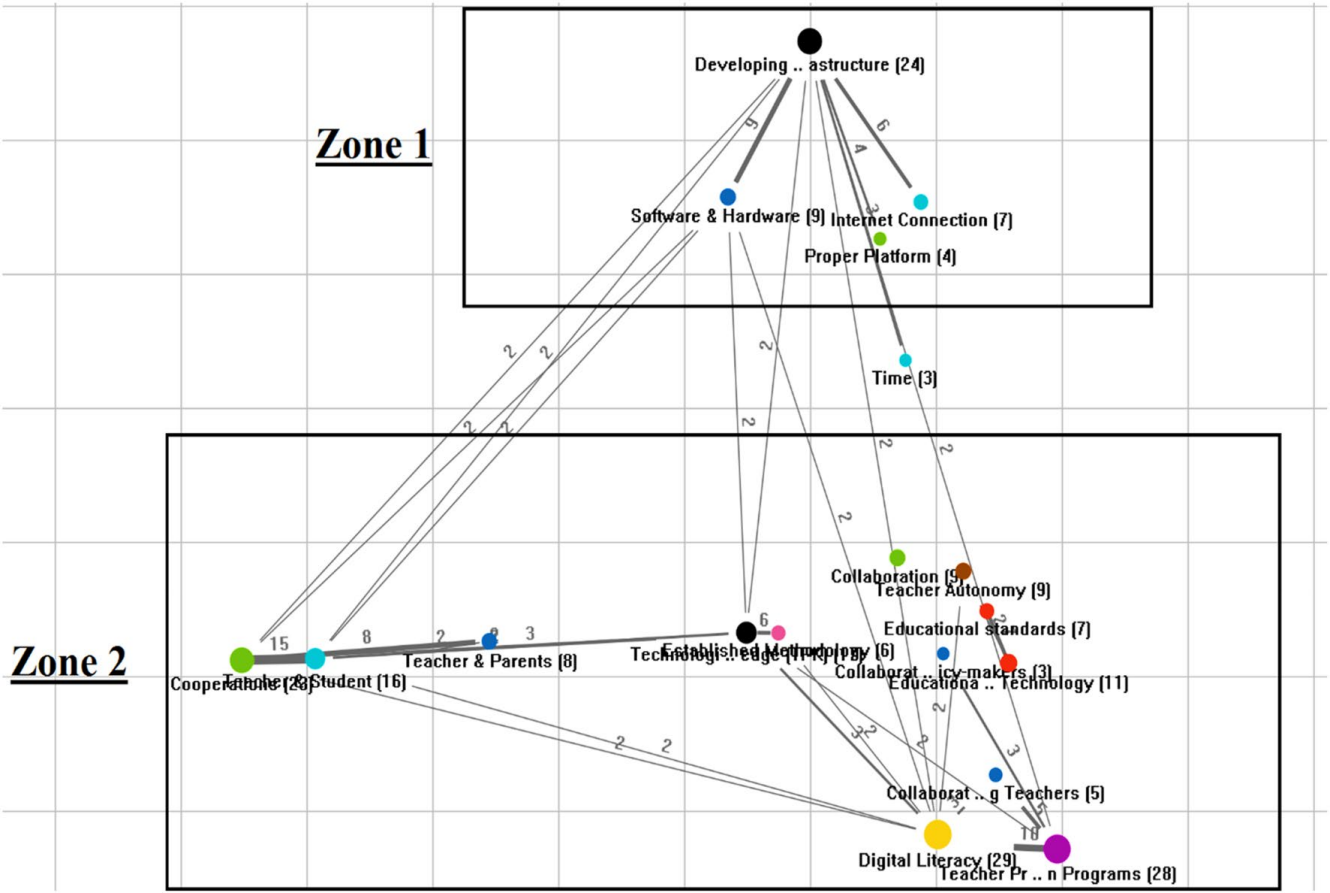
Code map of the EFL teachers’ suggestions

We used the Code Map of the themes to see how the EFL teachers’ suggestions concerning using CALL during the pandemic can be addressed. The Code Map shows which suggestions (themes) are closer to each other, so they should be considered together when a program is planned, or a policy is made. The Code Map is obtained through the frequency of co-occurrence of the codes. Thus, the more frequently two codes are assigned together, the closer they are on the map. Figure 7 shows the map.

As seen in Fig. 7, there are two major zones that L2 educators and policymakers can address to facilitate using CALL during the pandemic for the EFL teachers. Zone 1 highlights the infrastructure development. Here, the policymakers need to address infrastructure development concerning the time of the pandemic. Zone 2 indicates relationships between the two parent themes, cooperation, and teacher preparation program. Here, in zone 2, L2 educators need to revisit the CALL teacher preparation programs to not only address the sub-themes related to teacher preparation programs (e.g., developing digital literacy, collaboration among teachers) but also develop teachers’ knowledge on how to cooperate with parents and students during the pandemic. As the lines among the themes and sub-themes show in zone 2, such a CALL teacher preparation program needs to be multidimensional in that it can address different aspects of cooperation and teacher preparation programs interactionally.

## Discussion

This study was an attempt to explore the Iranian EFL teachers’ technological needs and their suggestions for using CALL during the COVID-19 pandemic. The findings indicated that EFL teachers believed that CALL teacher preparation programs should prioritize TPK, then TCK, and finally TK. Moreover, they stated that CALL teacher preparation programs should develop their knowledge concerning the intersections of TCK/TPK and TPK/TK. Furthermore, the study’s findings indicated that Iranian EFL teachers suggested that CALL can be used during the pandemic if cooperation among teachers, students, and parents will be made, technological-related infrastructures will be developed, and teacher preparation programs will develop EFL teachers’ collaboration, digital literacy, teacher autonomy, and TPK with special attention to the educational needs made by the pandemic situation.

The first research question was “[W]hat are Iranian EFL teachers’ technological needs to be addressed in CALL teacher preparation programs?” The findings highlighted that EFL teachers need to integrate technology into their classes effectively (TPK = 76.7%, TCK = 41.7%) rather than become technology experts (TK = 38.3%). This aligns with what Peters (2006) mentioned: teachers need to learn how to use technology, not the mere knowledge of technology types. Moreover, the interactions obtained among different components of TPACK, including TCK/TPK and TPK/TK, show that some components need to be revisited to be used in new CALL training models to develop teachers’ knowledge of integrating CALL in their classrooms. Thus, it can be argued that the TPACK model needs to be reformulated to become a context-oriented one, meaning that based on the primary needs of the EFL teachers in different contexts, the attention paid toward its components by the teacher educators and policymakers may change (Chapelle & Hegelheimer, 2004). Furthermore, such contextually-oriented TPACK may remove teachers’ lack of proper training as a key factor causing teachers’ negative perceptions toward integrating CALL in their classes (Chambers & Bax, 2006).

One reason concerning Iranian EFL teachers’ belief about prioritizing TPK, then TCK, and finally TK in CALL teacher education programs might be due to the ELT curriculum covered in Iran. Since EFL students at different educational levels, such as BA and MA, participated in CALL courses, they were instructed mostly about the materials, which are about TK. Therefore, they have already been informed about the necessary TK that helps them integrate technology in their classes. Relatively, such CALL courses help EFL teachers develop their TCK in that they are informed how to use technology to make content appropriate for their classes. However, these CALL courses delivered in the curriculum of BA and MA of TEFL pay the least attention towards TPK. Therefore, it will not be easy for the EFL teachers to understand how to address the pedagogical affordances and constraints of technology types in different pedagogical contexts.

The second research question of this study was “[W]hat are Iranian EFL teachers’ suggestions for using CALL during the COVID-19 pandemics?” The findings of this study indicated that one of the suggestions made by the EFL teachers to use CALL during the pandemic is cooperation among teachers, parents, and students. According to Chen et al. (2019), parents’ perceptions about using CALL are rather critical. It is without saying that “parents’ cultural backgrounds, educational experiences, and family’s socioeconomic status on students’ learning” (Chen et al., 2019, p. 2) can impact on using CALL. This is because parents are now involved in their children’s learning through the rapid technological innovations (Patrikakou, 2016) and have access to their children’s education through online learning involvement (Selwyn et al., 2011). Therefore, cooperation between teachers and parents can help reimage the influential role of parents in successful CALL during the pandemic. Furthermore, the same cooperation should be made between the teachers and students in which teachers can obtain students’ problems with using CALL (e.g., lack of the prerequisite infrastructure). By cooperating with parents and students, then, the teachers can use their critical thinking and their TPK, TCK, and TK to provide practical solutions for the problems caused by the pandemic.

Addressing CALL infrastructures was another suggestion made by the Iranian EFL teachers to make CALL more applicable during the pandemic. Overall, this is essential to innovate educational infrastructure to infuse technology into L2 classes (Le & Song, 2018). According to Garrett (2009), technological infrastructure is pivotal when educationalists want to make a relationship between pedagogy, theory, and technology. Thus, governments need to address the infrastructures when they want to transform face-to-face instructions into online instructions. If the necessary infrastructures are met, then the engagement of teachers and students will be developed. This way, problems such as students’ demotivation can be removed. In the COVID-19 situation, the role of education policymakers is critical to revisit some components related to infrastructure, for example, time. For the Iranian context in which the EFL teachers and students had not experienced the online classes before the pandemic, it is a critical question to be raised that “how much time is enough for each session of the classes?” Answering such questions and addressing other issues related to infrastructures, such as the internet connection, software and hardware, and proper platforms, may lead to prosperous online classes during the pandemic.

Finally, the findings showed that Iranian EFL teachers suggested CALL teacher preparation programs as a facilitator to make it more applicable to use CALL (Stockwell, 2009), especially during the pandemic. They believed that CALL teacher preparation programs could address teacher collaboration, digital literacy, teacher autonomy, and issues related to TPK, which are specified to the pandemic situation. This is critical to have CALL teacher preparation programs addressing collaboration among teachers “as apprenticeships in the practice of new technological knowledge or skills” (Hubbard & Levy, 2006, p. 304), especially to tackle educational issues during the pandemic. Due to the censorious nature of the COVID-19 pandemic, teachers need to collaborate with their colleagues to use the experiences of their critical friends. Thus, teacher preparation programs should provide the venue for collaboration among teachers. Moreover, the EFL teachers mentioned the role of CALL teacher preparation programs in developing teachers’ digital literacy to suggest using CALL during the pandemic. Addressing digital literacy in CALL teacher preparation programs can develop teachers’ digital identity (Engeness, 2021). By developing teachers’ digital identity, they can deal with the problems that happen during the pandemic. It is so since developing teachers’ digital identity makes teachers competent to use their critical thinking to cope with educational problems.

Furthermore, CALL teacher preparation programs should help teachers to develop their autonomy concerning using CALL. The rapid obsolescence of technology types asks for developing teachers’ autonomy to work with technology in different contexts (Torsani, 2016). The autonomous teachers would be able to select, evaluate, and implement technology types in their classes. Thus, it can be argued that by developing teachers’ autonomy through CALL teacher preparations, they can select, evaluate, and implement technology regarding the educational needs imposed by the pandemic. Also, addressing TPK related to the needs of the pandemic can be one of the crucial responsibilities of CALL teacher preparation programs through which teachers can identify the pedagogical affordance and constraint of the technology types in accordance with their context and develop proper pedagogical strategies to tackle the constraints (Koehler & Mishra, 2009), especially during the COVID-19 pandemic.

## Conclusion

The current qualitative study investigated the Iranian EFL teachers’ technological needs and their suggestions for using CALL during the COVID-19 pandemic. We analyzed the narratives produced by 66 EFL teachers for their themes through thematic analysis. The findings showed that CALL teacher preparation programs should prioritize technological pedagogical knowledge (TPK), then technological content knowledge (TCK), and finally technological knowledge (TK). Moreover, teachers stated that CALL teacher preparation programs should develop their knowledge concerning the intersections of TCK/TPK and TPK/TK. Furthermore, Iranian EFL teachers suggested that CALL can be used during the pandemic if cooperation among teachers, students, and parents will be made, technological-related infrastructures will be developed, and teacher preparation programs will develop EFL teachers’ collaboration, digital literacy, teacher autonomy, and TPK with special attention to the educational needs made by the pandemic situation.

Effective and responsive teacher preparation and professional development programs that intend to develop teachers’ best practices should be allied with teachers’ technological needs. It would be more effortless to develop teachers’ knowledge, literacies, and practices and mitigate their psychological impediments by clearly recognizing and addressing their needs and concerns. Thus, the findings of this study have valuable implications for various stakeholders in the context of foreign language education, including teacher educators, PD designers and providers, and decision-makers by highlighting promising directions to devote their precious time, energy, and resources. First, PD designers and providers should focus on the common knowledge and areas that many teachers find challenging, such as TPK, then TCK, and finally TK. Second, many teachers in this study perceived that the development of the intersections of TCK/TPK and TPK/TK require more attention. Finally, due to the pandemic situation and adopting new standards, teachers needed considerable PD support in developing their collaboration, digital literacy, and autonomy. Although the study conducted in the Iranian context and with Iranian EFL teachers, the exploratory nature of the study through which some suggestions proposed by the teachers can be investigated in the other contexts to find the overall universal suggestions concerning the topic.

In the end, like many other research in the field, the current study had some limitations. The study was performed on a sample of Iranian language teachers in which the generalization of the findings should be made with great care. As mentioned earlier, the participants have attended PD online workshops, and their needs might differ from those in other under-resourced contexts. Therefore, a comparative study between teachers’ technological needs with different underlying resources and identifying the potential divergence and convergence factors would be valuable.

## Data Availability

The data that support the findings of this study are available from the corresponding author on reasonable request.
